# Exosomal miR-182 derived from bone marrow mesenchymal stem cells drives carfilzomib resistance of multiple myeloma cells by targeting SOX6

**DOI:** 10.1186/s13018-023-04399-9

**Published:** 2023-12-07

**Authors:** Shifeng Long, Shengping Long, Honglei He, Liang Luo, Mei Liu, Ting Ding

**Affiliations:** Department of Hematology, The Affiliated Hospital of Jinggangshan University, No. 110, Jinggangshan Avenue, Jizhou District, Ji’an, 343000 Jiangxi Province People’s Republic of China

**Keywords:** Exosomal miR-182, Carfzomil resistance, SOX6, Bone marrow mesenchymal stem cells, Multiple myeloma

## Abstract

**Background:**

Multiple myeloma (MM) is a common hematological malignancy. Drug resistance remains to be a major clinical challenge in MM therapy. In this study, we aim to investigate the functional roles of bone marrow mesenchymal stem cells (BMSC)-derived exosomal miR-182 on the carfilzomib resistance of MM and its underlying mechanism.

**Methods:**

qRT-PCR and Western blot methods were utilized to confirm the gene or protein expressions. CCK-8 and transwell assays were performed to measure the capabilities of proliferation, migration, and invasion. The molecular interactions were validated through ChIP and Dual luciferase assay.

**Results:**

Our findings indicated that miR-182 expression was upregulated in serum, BMSCs and BMSC-derived exosomes from MM patients. Hypoxia-inducible factor-1α (HIF-1α), a key transcriptional factor in tumor microenvironment, could boost miR-182 expression by directly binding to its promoter, thus favoring exosomal secretion. Moreover, exosomal miR-182 from BMSCs could be transferred to MM cells and was able to promote malignant proliferation, metastasis, and invasion, as well as decrease the sensitivity of MM cells against carfilzomib. Additionally, SOX6 was identified as a downstream target of miR-182 in MM cells, and its expression was negatively regulated by miR-182. Rescue experiments proved that loss of SOX6 in MM cells dramatically reversed the promoting roles of BMSC-secreted exosomal miR-182 on proliferation, metastasis, and carfilzomib resistance in MM cells.

**Conclusion:**

Collectively, our findings indicated that exosomal miR-182 derived from BMSCs contributed to the metastasis and carfilzomib resistance of MM cells by targeting SOX6. This study sheds light on the pathogenesis of the BMSC-derived exosome containing miR-182 in the malignant behaviors of MM cells and carfzomib resistance.

## Highlights

- miR-182 is greatly elevated in serum, BMSCs, and BMSCs-exosomes derived from MM patients.
- HIF-1α binds to miR-182 promotor to activate its transcription and exosome secretion in BMSCs.
- SOX6 is a downstream target of miR-182 and is negatively regulated by miR-182.
- Exosomal miR-182 secreted by BMSCs promotes MM cell proliferation, metastasis, and carfilzomib resistance by targeting SOX6.

## Introduction

Multiple myeloma (MM) is a malignant tumor of bone marrow plasma cells, ranking second in the incidence of hematological malignancies, with a mortality rate accounting for 20% of all hematological malignancies in the US [1]. Chemotherapy remains one of the main treatment strategies for MM [2]. However, with the increase of the number of chemotherapy, the drug resistance rate of patients continues to rise, which has become a major bottleneck in the clinical treatment of MM [1]. Carfilzomib, approved by the FDA as the second proteasome inhibitor after bortezomib, is used to treat MM patients who have received at least two prior treatments, including bortezomib and immunomodulators [3]. Nevertheless, the current mechanisms of resistance to carfilzomib remain not entirely clear and need further investigation.

Growing evidences demonstrated that the bone marrow microenvironment (BMM) plays a significant role in the pathogenesis and drug resistance of MM [4]. BMM is composed of cellular and non-cellular elements, which can communicate with MM cells to provide material support for MM growth, metastasis, angiogenesis, immunosuppression and chemotherapy resistance [5]. Generally, the crosstalk between BMM and MM cells is mainly dependent on direct cell-to-cell contact and the generation of soluble factors such as extracellular vesicles (EVs) and various growth factors [6]. Exosomes are the most important form of EVs, comprising membrane structures that transport signaling molecules and serve as important mediators for intercellular communication [7]. Over the past few decades, exosomes have been proved to be responsible for the bidirectional transfer of proteins, lipids, and nucleic acids between the BMM and MM cells [8, 9]. Few studies demonstrated that BMMs-derived exosomes participated in malignant proliferation, stemness maintenance and drug resistance [10, 11]. Nevertheless, the detailed crosstalk between MM cells and BMM and their functional mechanism remains to be further examined.

MicroRNAs (miRNAs) are one of endogenous non-coding small RNA with lengths ranging from 18 to 22 bp [12]. For decades, miRNAs have attracted significant interests due to their ability to remain stable in various bodily fluids and their pivotal regulatory functions in gene expression [13–17]. Currently, miRNAs are well-known to be involved in MM pathogenesis and progression [18]. MiR-182-5p (miR-182) is a member of the miR-183/miR-96/miR-182 cluster, and was proved to be widely expressed in exosomes derived from various cancers, such as breast cancer, prostate cancer, glioma, and oral squamous cell carcinoma, and served as an oncogenic role to promote tumor development [19–22]. A previous study showed that miR-182 promoted cell adhesion-mediated resistance to doxorubicin in MM by targeting PDCD4 [23]. Moreover, miR-182 was proved to be enriched in BMSC-secreted exosomes [24–26], and its expression level was closely correlated with hypoxia induction [27]. Based on this evidence, we speculated that miR-182, secreted by BMSCs, could be one of the factors contributing to carfilzomib resistance in MM under hypoxic conditions.

Our preliminary data from biological information analysis revealed a binding site for miR-182 in the 3′ untranslated region (3′-UTR) of the SRY-Box transcription factor 6 (SOX6), indicating a potential binding relationship between miR-182 and SOX6. Interestingly, the previous reports confirmed that SOX6 was downregulated in MM patients, and its overexpression greatly repressed MM cell proliferation and induced apoptosis [28]. Furthermore, SOX6 showed a significant involvement in tumor chemotherapy resistance in cervical cancer [29]. Thus, we hypothesized that under hypoxic condition, BMSCs-derived exosomal miR-182 promoted MM cell migration, invasion, and carfilzomib resistance by targeting SOX6.

## Materials and methods

### Clinical sample collection

Twelve patients diagnosed with MM were enrolled at The Affiliated Hospital of Jinggangshan University Hospital. The diagnosis criteria and treatment efficacy were based on the International Myeloma Working Group. All participants were primary and untreated MM patients. In the control group, ten patients with benign diseases (BD), including iron deficiency anemia and thrombocytopenia, were chosen in this work. Bone marrow (BM) samples were collected from the iliac bones of newly diagnosed MM and BD patients. Approximately 3–4 ml of BM and 5 ml of peripheral blood samples were obtained from all subjects. All procedures were conducted under sterile conditions. The collection and use of clinical specimens were approved by the ethics committee of The Affiliated Hospital of Jinggangshan University. All subjects were provided with written informed consent.

### Bone marrow-derived mesenchymal stem cells (BMSC) isolation

BM mononuclear cells were isolated using human BM mononuclear lymphocytes separating medium (Biological Products Technology; Tianjin Haoyang; China). The process involved slowly adding BM into the separating medium, followed by centrifugation at 1000 × g for 20 min at 20 °C, using slow acceleration and deceleration settings. The primary BMSCs were then cultured and selected in plastic flasks using the adherence method and were utilized at the third to fourth passage.

### BMSC characterization

To identify phenotypic surface markers of BMSCs, flow cytometry was employed with specific antibodies including PE-conjugated CD29 (ab218273, Abcam, Cambridge, MA, USA), CD73 (ab282789, Abcam), CD105 (ab231774, Abcam), CD11b (ab213186, Abcam), CD34 (ab134207, Abcam), and CD45 (ab269297, Abcam) antibodies. Appropriate isotype controls were utilized. Data analysis was performed using FlowJo 10.6.2 software (FlowJo, LLC).

For osteogenic differentiation, BMSCs were induced using the osteogenic differentiation medium kit (Thermo Fisher Scientific, Waltham, MA, USA). The osteogenic differentiation complete medium was prepared according to the kit instructions, and the culture medium was changed every 3 days. After the induction for 21 days, the osteogenic differentiation was assessed and analyzed using alizarin red staining.

For lipogenic differentiation, BMSCs were induced using the lipogenic differentiation medium kit (Cyagen, CA, USA). Lipogenesis-induced differentiation A and B solutions were prepared following the kit instructions until large and round lipid droplets appeared. After 21 days, Oil Red O staining was used to detect lipid droplets.

### Exosome isolation

Exosome isolation was carried out using the ExoQuick-TC system following the instructions from System Bioscience. Briefly, BMSCs were cultured in cell culture plates until reached 70% confluence. Then, the cells were washed with phosphate-buffered saline (PBS, Gibco) and incubated with DMEM containing 10% exosome-depleted FBS (Gibco) for 24 h. After incubation, the DMEM was collected and centrifuged at 2000 × g for 30 min. The supernatant was filtered through a 0.22 μm filter (Sigma-Aldrich, St Louis, MO, USA), and the exosome isolation reagent (Invitrogen) was added. The mixture was incubated overnight at 4 °C and then centrifuged at 10,000 × g for 1 h at 4 °C. The supernatant was removed, and the exosomes were resuspended in 100 μL PBS for next experiments.

### Exosome characterization

Characterization of the exosomes was performed using transmission electron microscopy (TEM), nanoparticle tracking analysis (NTA), and western blot. For TEM imaging, exosomes were fixed with 2.5% glutaraldehyde in calcium carbonate buffer (Sigma-Aldrich) for 1 h and negatively stained with 2% phosphotungstic acid (Sigma-Aldrich) for approximately 2 min. The samples were then imaged using a TEM (FEI Tecnai G2 Spirit, Thermo Fisher Scientific, Waltham, MA, USA) at 80 kV. For NAT analysis, exosomes in PBS were diluted to a concentration of approximately 0.05 μL/mL and injected into the sample chamber. The samples were imaged using the Nanosight NS 300 system (NanoSight Technology, Malvern, UK), and the images were analyzed using the NTA analytical Software (version 2.3).

### Hypoxia induction

To induce hypoxia, BMSCs were subjected to a low oxygen environment (0.2% O_2_) for 3 days. The incubation was carried out at a temperature of 37 °C, with 5% CO_2_ concentration and 90% humidity. The medium used during the hypoxia treatment was collected for subsequent experiments. To prevent reoxygenation and maintain hypoxic conditions, cell lysis for protein and RNA extraction was performed directly within the Hypoxic Workstation.

### Cell culture

The MM cell lines MM.1S and U266 were procured from the Cell Bank of the Chinese Academy of Sciences (Shanghai, China). These cell lines were cultured in Rosewell Park Memorial Institute (RPMI) medium 1640 (Invitrogen, Carlsbad, CA, USA). The culture medium was supplemented with 10% fetal bovine serum (FBS, Gibco, Grand Island, NY, USA) and 1% penicillin–streptomycin. The cells were maintained at a temperature of 37 °C in an environment with 5% CO_2_. For carfilzomib treatment, MM.1S and U266 cells were seeded into a 96-well plate at a density of 1 × 10^4^ cells per well. After 24 h of initial seeding, each well was treated with carfilzomib (Sigma-Aldrich) at concentrations ranging from 0.01 to 1000 nM.

### Exosome uptake experiment

The BMSC-derived exosomes were labeled with PKH26 Fluorescent dye (Sigma-Aldrich) using a previously published protocol [30]. The PKH26-labeled exosomes were added to MM cells for incubation for 24 h. Afterwards, MM cells were fixed with 4% PFA. MM cells were further stained with DAPI staining (Sigma-Aldrich) for 15 min. Fluorescent images were captured using a Zeiss LSM 710 laser scanning confocal microscope (Zeiss).

### Cell transfection

The miR-182 antagomir and miR-182 mimics, as well as their negative control (antogomir NC, mimics NC), were purchased from Biossci Company (Wuhan, China). The lentiviral constructs expressing HIF-1α (lv-HIF-1α) and empty lentiviral vectors (lv-NC) were obtained from Genepharma (Shanghai, China). Short hairpin RNAs targeting SOX6 (shSOX6) and control shRNA (shNC) were acquired from Genepharma. For cell transfection, miR-182 antagomir, lv-HIF-1α and their negative controls (lv-NC, antagomir NC) were transfected into BMSCs by using Lipofectamine 2000 (Invitrogen). ShSOX6, miR-182 mimics and their NCs (mimics NC, shNC) were transfected with MM cells by using Lipofectamine 2000 (Invitrogen). 48 h later, cells were collected for the measurement of transfection efficiency.

### Real-time polymerase chain reaction (qRT-PCR)

Total RNA was extracted using the Trizol reagent (Invitrogen). RNA (1 µg) was subjected to reverse transcription using the RNA reverse transcription kit (Takara, Osaka, Japan). Then, the synthetic cDNA was used for qRT-PCR assay using the SYBR Premix Ex Taq (Takara, Osaka, Japan) based on the manufacturer's instructions. Primer sequences for the qRT-PCR were provided as follows: miR-182 forward primer: 5′- -CTCTGTGTAAACGGGTCCTCGACTG -3′, reverse primer: 5′-TCCGGGTGTCGTGGAGTCG-3′; HIF-1α forward primer: 5′- CAGCTATTTGCGTGTGAGGA -3′, reverse primer: 5′- CCTCATGGTCACATGGATGA -3′; SOX6 forward primer: 5′-AGCTTCGGATTGGGGAGTAT-3′, reverse primer: 5′- GAGGCGATGGTGTGGTAGTT-3′; GAPDH forward primer: 5′- GCAAGTTCAACGGCACAG-3′, reverse primer: 5′-GCCAGTAGACTCCACGACAT-3′. U6 forward primer: 5′-GCTTCGGCAGCACATATACTAAAAT-3′, reverse primer: 5′- -CGCTTCAGAATTTGCGTGTCAT-3′. 2^−△△Ct^ method was employed to calculate relative RNA expression. The mRNA expressions were normalized to the GAPDH, and miRNA expression was normalized to U6.

### Western blot

Cellular protein was isolated using a radio-immunoprecipitation assay buffer supplemented with protease inhibitors. Subsequently, the protein concentrations were determined using a BCA protein assay (Thermo Fisher Scientific). Then, 30 μg of protein was separated via SDS-PAGE and then transferred onto polyvinylidene fluoride membranes (Merck Millipore, MA, USA). To ensure specificity and accurate detection, the membranes were blocked and washed with PBS before being exposed to primary antibodies against specific proteins, including CD9 (ab236630, 1:1000, Abcam), TSG101 (ab125011, 1:1000, Abcam), Calnexin (ab22595, 1:1000, Abcam), SOX6 (ab64946, 1:1000, Abcam), and GAPDH (ab8245, 1:2000, Abcam). Following primary antibody incubation, the membranes were once again washed with PBS and subsequently exposed to secondary antibodies (A-11001, 1:2000, Invitrogen) for 1 h. Finally, an ECL kit (Merck Millipore) was utilized to visualize the protein bands. Image J software was used for quantifying band intensity.

### Cell counting Kit-8 (CCK-8) assay

CCK-8 kit (Boster Biological Technology Co., Ltd, China) was used for the examination of cell viability. Briefly, treated MM.1S and U266 cells were subjected to CCK-8 solution (10 µL/wells) and incubated for 2 h. After the incubation period, the absorbance at 450 nm was measured using a microplate reader (Thermo Fisher Scientific). Based on the obtained results, the IC50 value for carfilzomib-treated samples was calculated using GraphPad Prism 5.0 software.

### Chromatin immunoprecipitation (ChIP) assay

BMSCs were harvested and subjected to treatment with RIP lysis buffer obtained from Merck Millipore. Following this, cell cross-linking and sonication procedures were carried out. To the samples, 900 μl of ChIP Dilution Buffer, 20 μl of 50 × PIC (protease inhibitor cocktail), and 60 μl of Protein A Agarose/Salmon Sperm DNA were added. After incubation, the samples were centrifuged, and the supernatant was carefully transferred to a separate tube. Subsequently, 1 μl of HIF-1α antibody (ab308433, 1:200, Abcam) or IgG antibody (ab205718, 1:1000, Abcam) was introduced to the supernatant, which was then incubated overnight at 4 °C to allow for immunoprecipitation. After the precipitation and subsequent washing steps, 1 μl of RNase A was added to each tube, followed by incubation at 37 °C for 1 h to remove any RNA contaminants. To each tube, 10 μl of 0.5 M EDTA, 20 μl of 1 M Tris–HCl, and 2 μl of 10 mg/mL proteinase K were added, and the samples were further incubated at 45 °C for 2 h. The DNA samples were then collected and quantified using agarose gel electrophoresis.

### Dual-luciferase reporter assay

The promotor sequences of miR-182 with wild-type (WT) binding site or mutant (MUT) site of HIF-1α were amplified and inserted into pGL3.0 vectors (Promega, Madison, WI, USA). For validating the interaction between miR-182 promoter and HIF-1α, the above recombinant luciferase vectors were co-transfected with lv-NC or lv-HIF-1α vectors into BMSCs by using Lipofectamine 2000 (Invitrogen). To verify the binding relationship between SOX6 3′-UTR and miR-182, we constructed a WT or MUT luciferase vector (pmirGLO vector, Promega) expressing SOX6 3′-UTR fragment with/without binding site of miR-182, called WT-SOX6 and MUT-SOX6. Then, WT-SOX6 and MUT-SOX6 luciferase vectors along with miR-182 mimics or mimics NC were transfected into MM cells by Lipofectamine 2000 (Invitrogen). After 48 h of incubation, luciferase activities were assessed using the Dual-Luciferase Reporter Assay Kit (Promega).

### Transwell assay

The treated MM cells were pre-starved for 6 h and followed by prepared-to-cell suspension (1 × 10^5^/mL) using a serum-free medium. For evaluating the capabilities of cell migration and invasion, a transwell chamber (8 µm, Corning Inc., Corning, NY, USA) was used. 200 μL of cell suspension was inoculated into the upper chamber, and 600 μL of complete medium was added in the bottom chamber. 24 h later, the un-migrated cells were removed and the bottom cells were washed and fixed with 4% paraformaldehyde for 10 min, followed by staining with 0.1 crystal violet for 5 min. Finally, the stained cells were imaged using a microscope (Olympus). The upper transwell chamber was pre-coated with Matrigel gel, and the invasion assay was conducted in line with the steps of the migration assay.

### Statistical analysis

In this study, data analysis was conducted using GraphPad Prism 5.0 software. The presented data is expressed as the mean ± standard deviation and was obtained from a minimum of three independent experiments. To compare the two groups, an unpaired Student's t-test was applied. For comparisons involving more than two groups, one-way analysis of variance (ANOVA) followed by the Tukey post hoc test was utilized. Statistical significance was considered at a significance level of *P* < 0.05.

## Results

### Isolation and identification of BMSCs

**Fig. 1:**
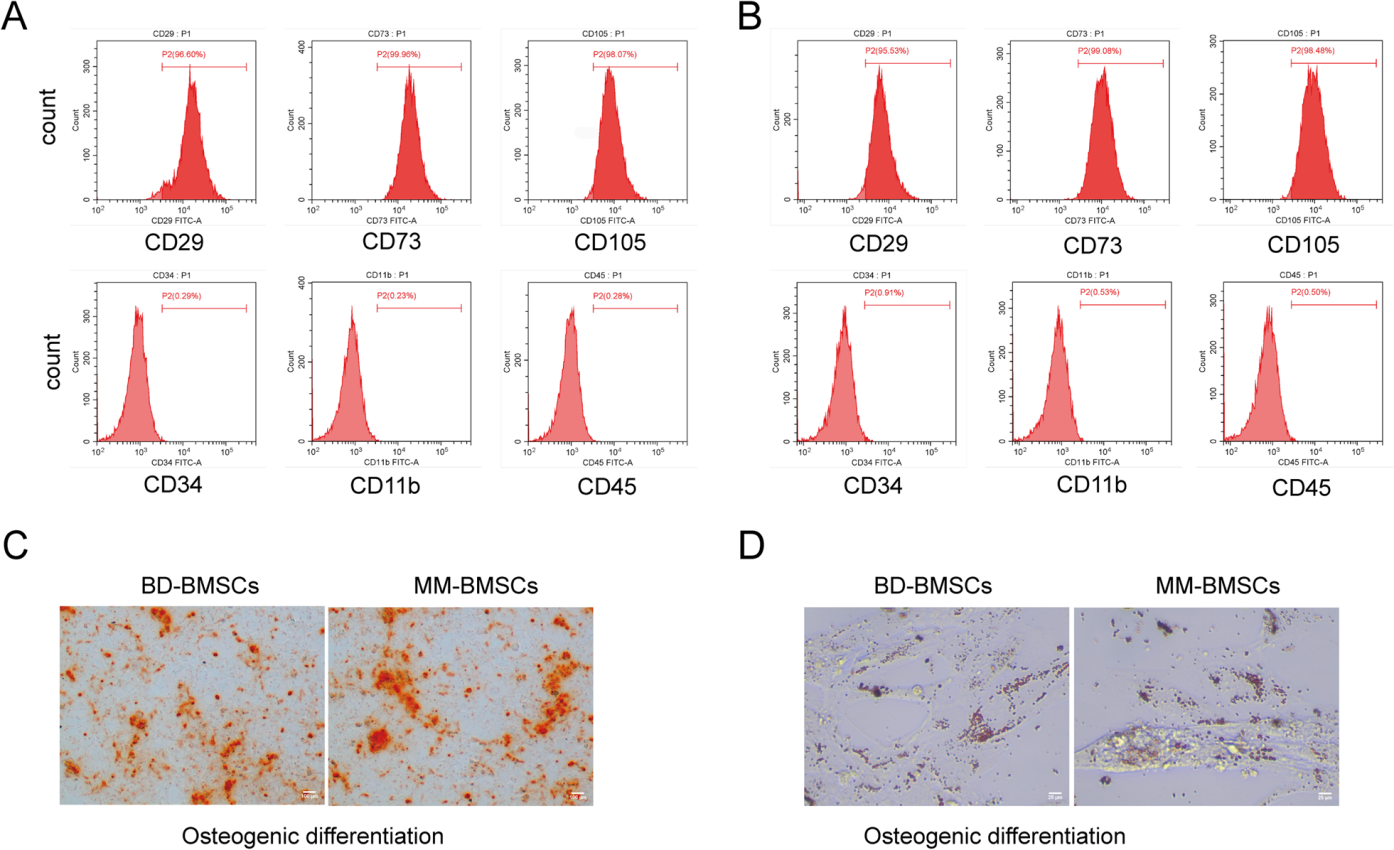
Isolation and identification of BMSCs. The BMSCs samples were isolated from the bone marrow of 12 cases of MM patients and 10 cases of BD patients. **A**, **B** Surface markers of bone marrow-derived mesenchymal stem cells isolated from **A** BD patients (BD-BMSCs) or **B** MM patients (MM-BMSCs) were examined by flow cytometry. **C** BD-BMSCs and MM-BMSCs were used for osteogenic differentiation induction, and the Alizarin red staining was performed to assess osteogenesis. **D** After lipogenic differentiation for 21 days, Oil red O staining determined the adipose differentiation ability

We isolated BMSCs from both BD and MM patients. The characteristics of the BMSCs were determined by analyzing the surface markers and differentiation capabilities. Flow cytometry data indicated that both BD-derived BMSCs (BD-BMSCs) and MM-derived BMSCs (MM-BMSCs) exhibited positive expressions of CD29, CD73, and CD105, and negatively expressed with CD45, CD34, and CD11b (Fig. 1A–B). In terms of osteogenesis induction, primary cultured BMSCs formed small lamellar mineralized nodules after 21 days of culture. Interestingly, both BD-BMSCs and MM-BMSCs showed significant bone calcification nodules, indicating osteogenic differentiation (Fig. 1C). Furthermore, after lipogenesis induction, Oil Red O staining revealed a significant presence of granular red lipid droplets in BMSCs isolated from MM and BD patients **(**Fig. 1D). Altogether, we have successfully isolated BMSCs from BD and MM patients.

### miR-182 was greatly elevated in serum, BMSCs, and BMSCs-Exos derived from MM patients

**Fig. 2:**
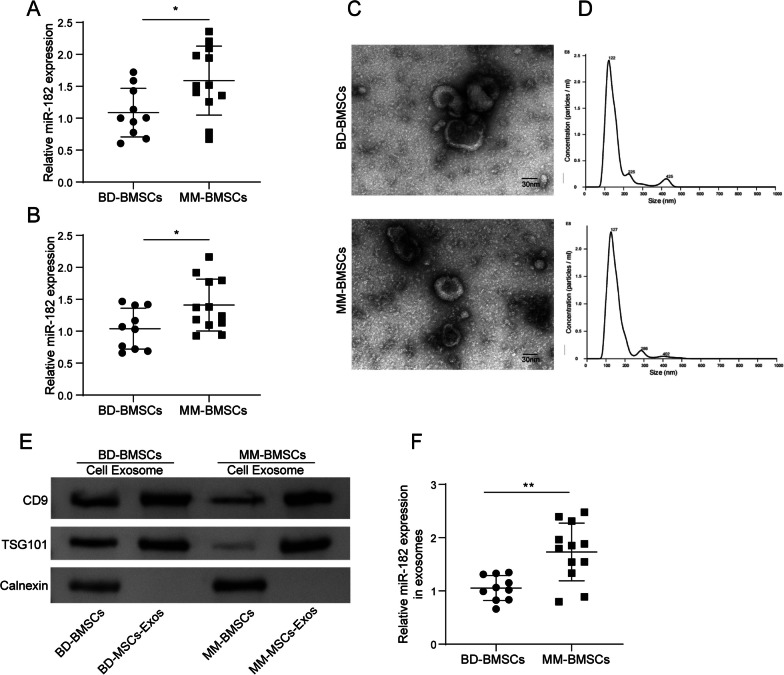
miR-182 was greatly elevated in serum, BMSCs, and BMSCs-Exos from MM patients. **A** Serum miR-182 level in 12 cases of MM patients and 10 cases of BD patients was detected by qRT-PCR. **B** The expression of miR-182 in BMSCs from BD patients (n = 10) and MM patients (n = 12) was determined using qRT-PCR. **C** TEM analysis revealed the characteristic cup-shaped morphology of exosomes. **D** NTA analysis for measurement of exosome diameter. **E** Western blot analysis confirmed the presence of exosome-related markers including CD9, TSG101 and Calnexin. **F** The expression of miR-182 in exosomes derived from BD-BMSCs and MM-BMSCs was quantified using qRT-PCR

Next, we investigated the expression level of miR-182 in the serum of both BD and MM patients. The results showed a significant elevation of miR-182 level in serum and BMSCs samples from MM patients compared to those from BD patients (Fig. 2A–B). Then, exosome samples were isolated from MM-BMSCs (MM-BMSCs-Exos) and BD-BMSCs (BD-BMSCs-Exos), and next characterized using TEM, NTA, and western blot analyses. The BMSCs-Exos displayed a typical rounded shape, with an average diameter of 50–160 nm (Fig. 2C–D). Additionally, specific exosome-related markers CD9 and TSG101 proteins were positively expressed in BMSCs-Exos, while Calnexin was negatively expressed (Fig. 2E). Moreover, the expression level of miR-182 in BMSCs-Exos was validated by qRT-PCR analysis. Data revealed a higher level of miR-182 in MM-BMSCs-Exos compared to that in BD-BMSCs-Exos (Fig. 2F). The above findings suggested that miR-182 exhibited a significant upregulation in the serum, BMSCs, and BMSC-Exos samples obtained from MM patients.

### The increase of miR-182 in BMSCs-Exos was closely correlated to hypoxia induction

**Fig. 3:**
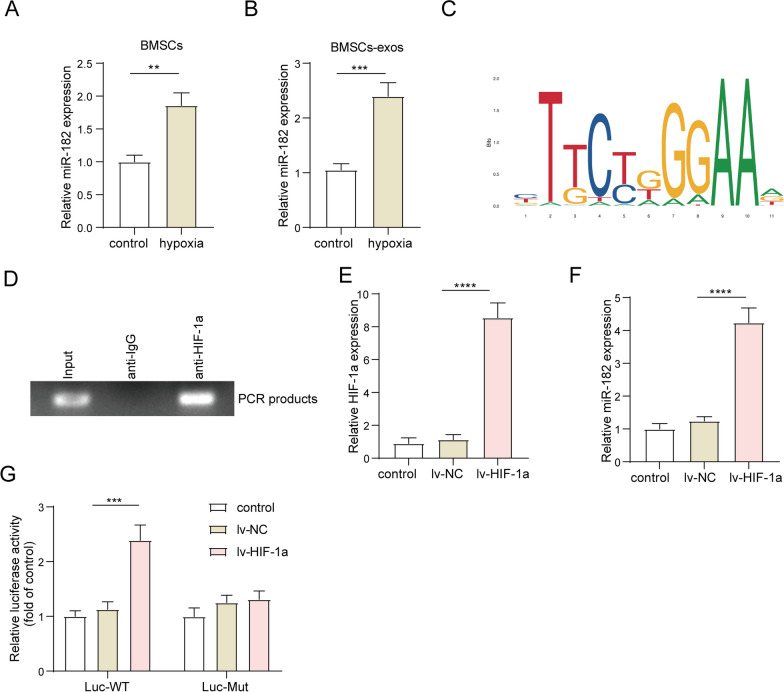
The increase of miR-182 in BMSCs-Exos was closely correlated to hypoxia induction. BD-BMSCs were cultured under normoxic or hypoxic conditions. **A**, **B** qRT-PCR was performed to detect the expression of miR-182 in **A** BD-BMSCs and **B** BD-BMSCs secreted exosomes. **C** Image of HIF-1α motif. **D** ChIP assay was conducted to examine the binding relationship between HIF-1α and miR-182 promoter. BD-BMSCs were transfected with lv-HIF-1α or lv-NC, respectively. **E**, **F** qRT-PCR was used to detect the expression of **E** HIF-1α and **F** miR-182. **G** The dual luciferase assay was employed to investigate the impact of HIF-1α on the transcriptional activity of miR-182

Previous study has revealed that the secretion of exosomal miR-182 from BMSCs could be induced under hypoxia conditions [27]. Considering that the microenvironment of MM was typically characterized by hypoxia, we decided to further investigate the impact of hypoxia on the expression and secretion of miR-182. As shown in Fig. 3A–B, hypoxia treatment greatly elevated miR-182 expression in BD-BMSCs and BD-BMSCs-Exos. HIF-1α was a critical effector of hypoxia induction, and its binding motif was presented in Fig. 3C. Through analysis by the Jaspar database, the potential binding site between HIF-1α and miR-182 promoter was found, and subsequently validated by ChIP and luciferase assays. As shown in Fig. 3D, it was observed a significant enrichment of miR-182 promoter in complexes precipitated by HIF-1α antibody (Fig. 3D). Next, HIF-1α overexpressing BD-BMSCs were constructed by transfection with lv-HIF-1α. Data was described that lv-HIF-1α transfection enhanced HIF-1α and miR-182 expressions (Fig. 3E–F**)**. Dual-luciferase assay indicated that HIF-1α overexpression significantly increased the luciferase activities of cells expressing WT-luciferase vector and had little impact on MUT-luciferase vectors (Fig. 3G). Taken together, these findings illustrated that hypoxia induction greatly promoted the transcription and exosome secretion of miR-182 in BMSCs.

### BMSCs-Exos-containing miR-182 could be absorbed by MM cells

**Fig. 4:**
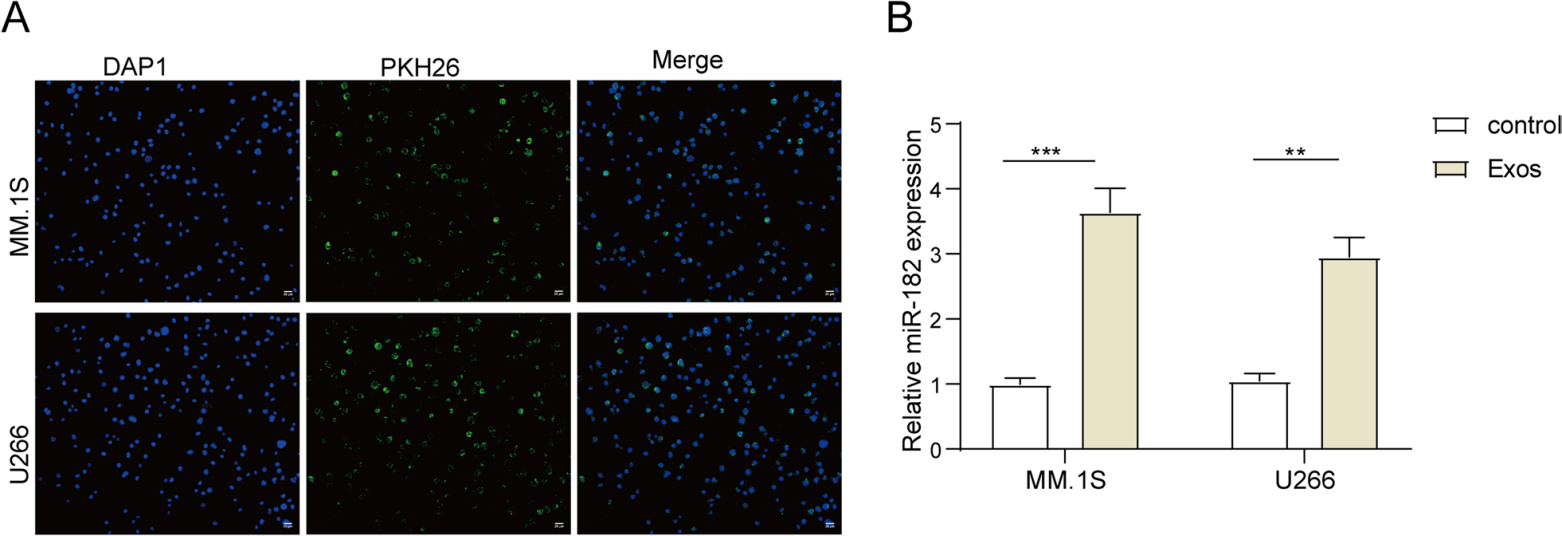
BMSCs-Exos-containing miR-182 could be absorbed by MM cells. MM.1S and U266 cells were treated with MM-BMSCs-Exos (50μg) for 24 h. **A** PKH26 staining was used to assess the uptake of MM-BMSCs-Exos by MM cells. **B** qRT-PCR was employed to measure the expression level of miR-182 in MM cells

MM.1S and U266 cells were co-cultured with MM-BMSC-Exos for 24 h. As depicted in Fig. 4A, PKH26 labeled MM-BMSCs-Exos were observed in MM.1S and U266 cells, indicating MM cells could absorb exogenous exosomes from MM-BMSCs. Moreover, the addition of MM-BMSC-Exos resulted in an elevation of miR-182 expression in MM.1S and U266 cells (Fig. 4B). These findings suggest that miR-182-containing BMSCs-Exos could be internalized by MM cells.

### Exosomal miR-182 secreted by BMSCs conferred the proliferation, metastasis, and carfilzomib resistance

**Fig. 5:**
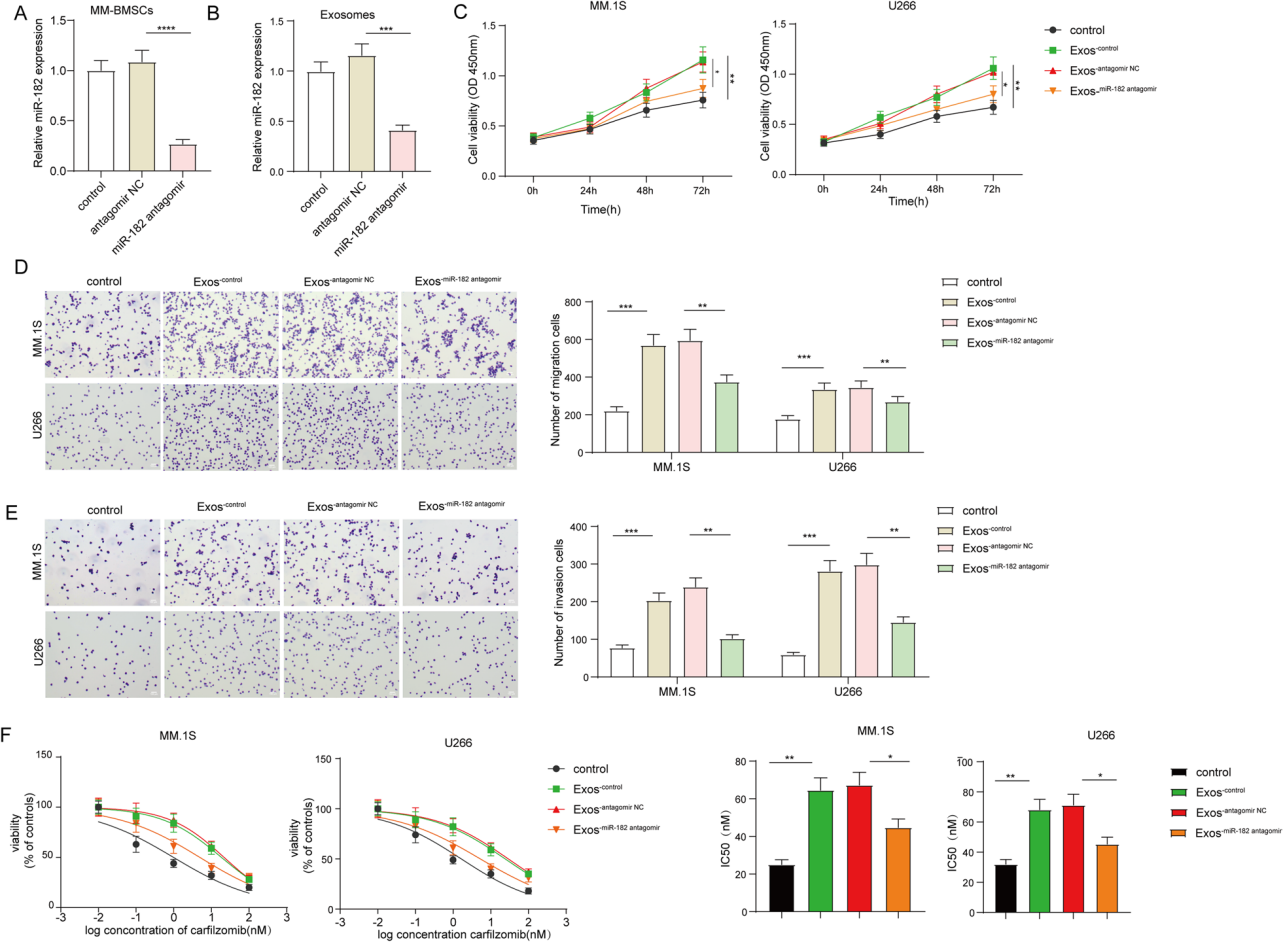
Exosomal miR-182 secreted by BMSCs conferred the proliferation, metastasis, and carfilzomib resistance. MM-BMSCs were transfected with antagomir NC or miR-182 antagomir, respectively. **A**, **B** qRT-PCR detected the expression of miR-182 in **A** MM-BMSCs and **B** MM-BMSCs-Exos. The exosomes were obtained from BMSCs that were transfected with antagomir NC or miR-182 antagomir, and named control, Exos^−control^, Exos^−antagomir NC^, Exos^−miR−182 antagomir^. **C** CCK-8 assay was utilized to evaluate MM.1S and U266 cell proliferation. **D**, **E** Transwell assay was performed to assess cell **D** migration and **E** invasion. **F** CCK-8 assay was conducted to determine the half-maximal inhibitory concentration (IC50) of carfilzomib

To further investigate the regulatory role of exosomal miR-182 derived by MM-BMSCs on biological behaviors of MM cells, we performed a knockdown of miR-182 in MM-BMSCs by transfection them with miR-182 antagomir. The results showed that the expression of miR-182 in both MM-BMSCs and MM-BMSCs-Exos was reduced upon miR-182 antagomir transfection (Fig. 5A–B). Next, the CCK-8 assay demonstrated that the treatment of MM-BMSCs-Exos significantly increased MM.1S and U266 cell viability than the control group, while this effect was greatly attenuated upon miR-182 was reduced in MM-BMSCs-Exos (Fig. 5C). Moreover, it also observed that the migration and invasion of MM cells were enhanced by MM-BMSCs-Exos, but these effects were attenuated when miR-182 was decreased in MM-BMSCs-Exos (Fig. 5D–E). To investigate whether exosomal miR-182 was involved in carfilzomib resistance in MM cells, the sensitivity of MM cells to carfilzomib was measured. The addition of MM-BMSCs-Exos significantly increased the carfilzomib IC50 value in both MM.1S and U266 cells, while this change was diminished by miR-182 inhibition in MM-BMSCs-Exos (Fig. 5F). In summary, the exosomal miR-182 secreted by BMSCs promoted proliferation, metastasis, and carfilzomib resistance in MM cells.

### SOX6 was a downstream target of miR-182

**Fig. 6:**
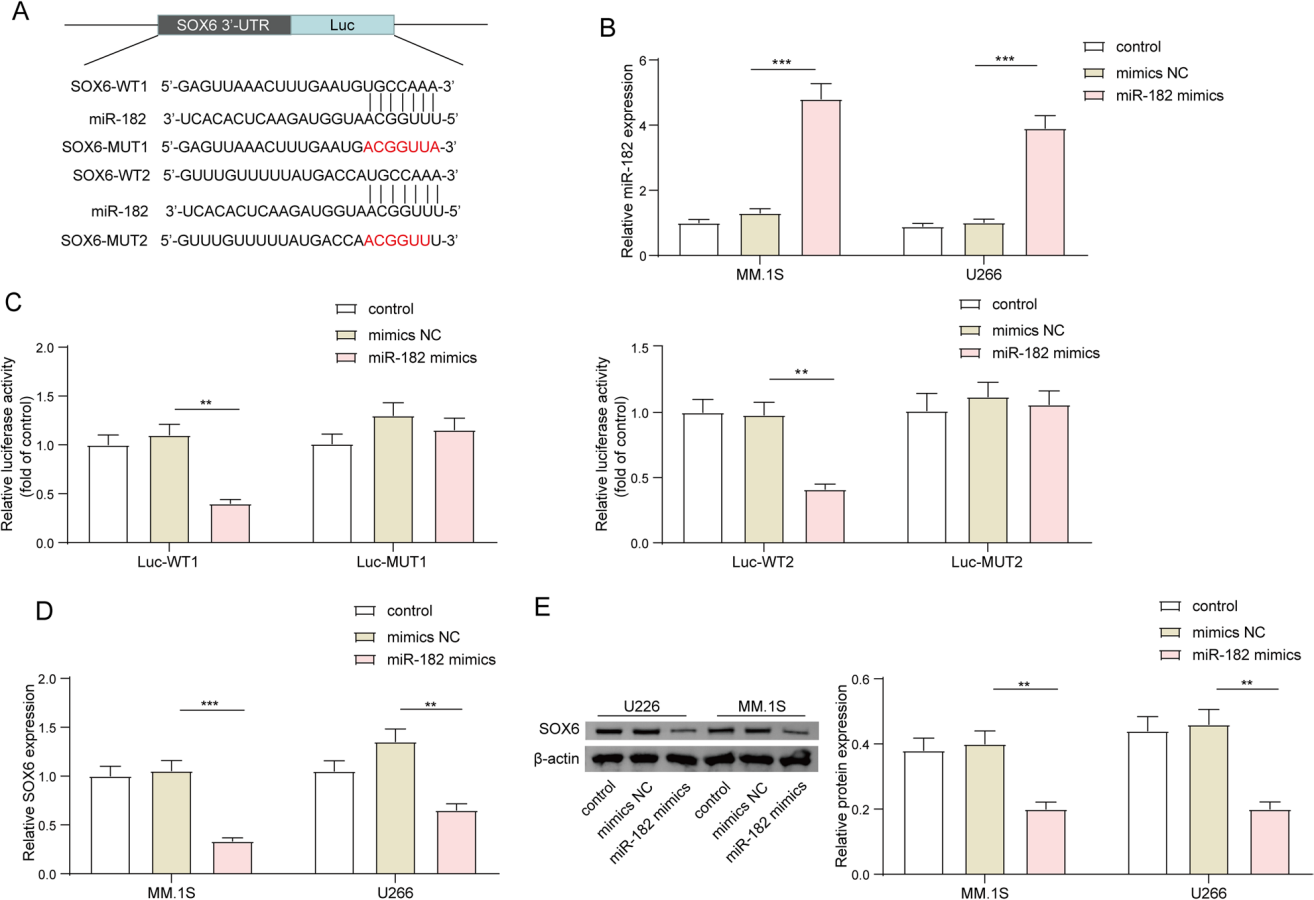
SOX6 was a downstream target of miR-182. **A** miRDB was used to predict the binding relationship between miR-182 and 3′-UTR regions of SOX6. MM.1S and U266 cells were transfected with miR-182 mimic or with mimics NC. **B** miR-182 expression was detected in MM.1S and U266 cells by qRT-PCR. **C** The binding relationship between miR-182 and SOX6 3′-UTR was examined using the dual-luciferase assay. **D**, **E** qRT-PCR and western blot were performed to quantify the gene and protein expression levels of SOX6

To explore the downstream mechanism of miR-182 in MM cells, the online prediction tool miRDB (http://www.mirdb.org/) was used to predict the potential target of miR-182. As shown in Fig. 6A, there were two potential binding sites of miR-182 on the 3′-UTR region of SOX6 (Fig. 6A). Next, we overexpressed miR-182 in MM.1S and U266 cells by transfecting with miR-182 mimics. The results showed that miR-182 mimics transfection significantly boosted miR-182 expression (Fig. 6B). Dual luciferase assay described that overexpression of miR-182 resulted in a significant reduction of luciferase activity in cells containing WT-SOX6 vectors at site1 and site2, while it did not affect the luciferase activity in cells expressing MUT-SOX6 vector at site1 and site2 (Fig. 6C). qRT-PCR and western blot assay showed that overexpression of miR-182 repressed the expression of SOX6 at mRNA and protein levels in MM.1S and U266 cells (Fig. 6D–E). In conclusion, these findings indicated that SOX6 was a downstream target of miR-182 and could be negatively regulated by miR-182.

### Loss of SOX6 restrained the biological roles of miR-182 in BMSCs-Exos

**Fig. 7:**
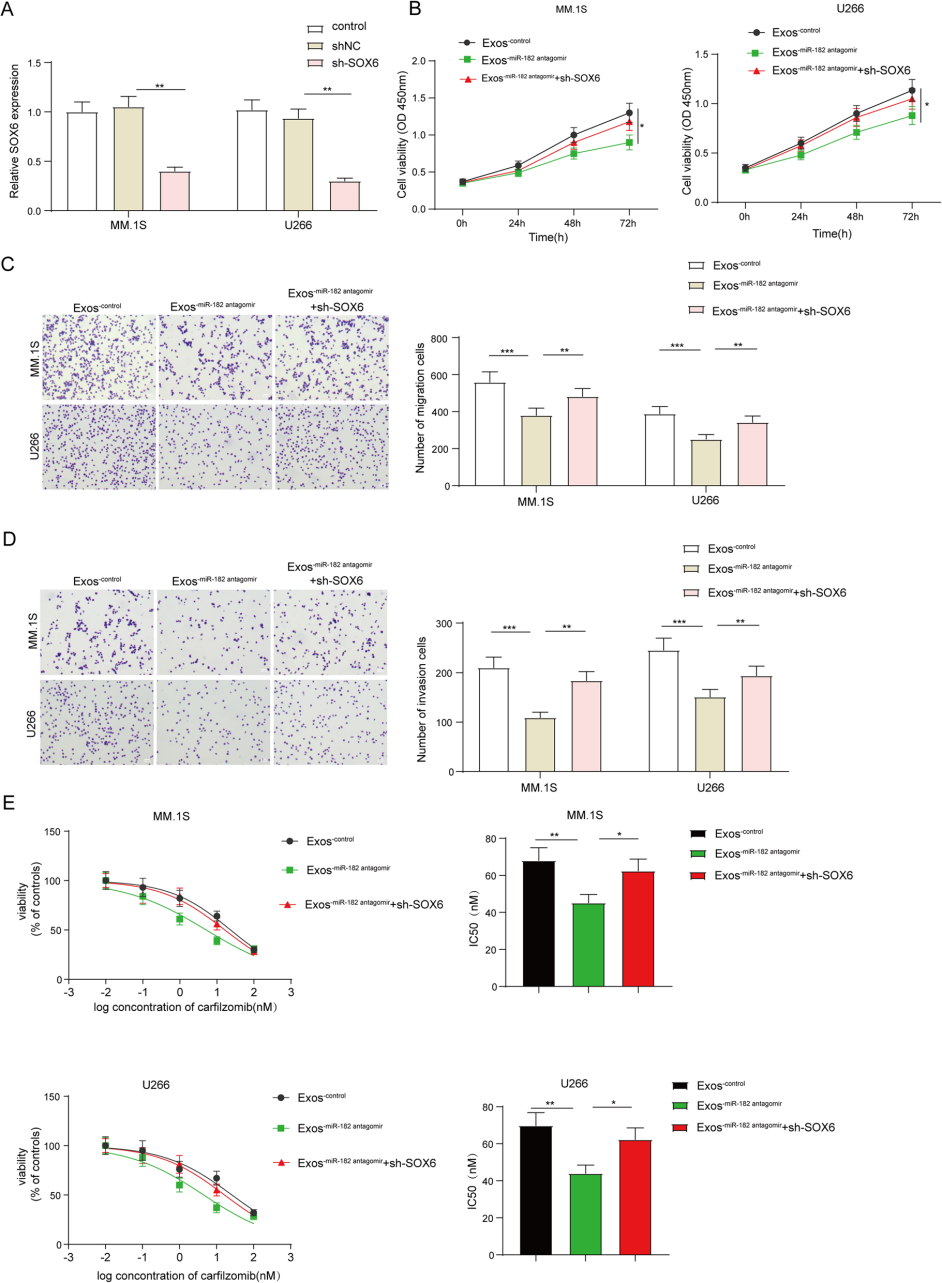
Loss of SOX6 restrained the biological roles of miR-182 in BMSCs-Exos. **A** MM.1S and U266 cells were transfected with shNC or shSOX6 for 48 h, then the transfection efficiency was determined by qRT-PCR. MM.1S and U266 cells were treated with Exos^−control^, Exos^−antagomir NC^, Exos^−miR−182 antagomir^ and Exos^−miR−182 antagomir^ + shSOX6. **B** CCK-8 assay was utilized to evaluate MM.1S and U266 cell proliferation. **C**, **D** Transwell assay was performed to assess MM.1S and U266 cell **C** migration and **D** invasion. **E** CCK-8 assay was conducted to determine the IC50 of carfilzomib

Finally, SOX6 was silenced in MM.1S and U266 cells to establish SOX6 downregulated cell lines. qRT-PCR results described that sh-SOX6 transfection remarkably reduced SOX6 expression (Fig. 7A). CCK-8 assay confirmed that compared to the Exos^−control^ group, the inhibitory role of miR-182 Exos^−antagomir^ was dramatically reversed by SOX6 inhibition (Fig. 7B). Consistently, the promoting effects of miR-182 Exos^−antagomir^ on MM.1S and U266 cell migration and invasion were diminished upon the loss of SOX6 (Fig. 7C–D). Compared to the Exos^−control^ group, it also observed that the repressive roles of Exos^−miR−182 antagomir^ on drug resistance of MM cells against carfilzomib were greatly overturned by SOX6 knockdown (Fig. 7E). Collectively, SOX6 was a direct target of exosomal miR-182 derived from MM-BMSCs.

## Discussion

Studies indicated that the high metastatic potential of MM is closely associated with treatment resistance [31]. One such agent used in MM treatment is carfilzomib, a proteasome inhibitor known for its potent anti-myeloma effects [32]. However, despite its initial efficacy, resistance to carfilzomib remains a significant clinical challenge in MM management. The study highlighted that BMSC-derived exosomal miR-182 significantly increased miR-182 expression within the MM cells, resulting in enhanced MM cell growth, metastasis, and resistance to carfilzomib. We also demonstrated that HIF-1α directly interacted to miR-182 promoter, potentially contributing to its increased expression under hypoxic condition. Furthermore, the downstream target of miR-182, SOX6, effectively countered the promotional effects of BMSC-derived exosomal miR-182 on MM progression and carfilzomib resistance. The study offered significant insights into the molecular mechanisms involved in BMSC-derived exosomal miR-182-regulated drug resistance, emphasizing miR-182 was a potential therapeutic target for MM treatment.

BMSCs are essential components of the BMM and play a crucial role in the development and progression of MM [33]. The dynamic crosstalk between BMSCs and MM cells influences disease behavior and treatment response [34]. Emerging evidence suggests that EVs, particularly exosomes, are critical mediators of intercellular communication between BMSCs and MM cells [35]. These exosomes carry various bioactive molecules, including miRNAs, that can modulate MM cell behavior and contribute to disease pathogenesis and drug resistance [35]. For instance, BMSC-derived exosomal miR-155 was shown to inhibit MM cell apoptosis while promoting MM cell division, stemness maintenance, and drug resistance [11]. Another study reported that the levels of exosomal miRNAs, specifically miR-16-5p, miR-15a-5p, miR-20a-5p, and miR-17-5p, were notably reduced in circulating exosomes of bortezomib -resistant MM patients, indicating a potential correlation between these miRNAs and bortezomib resistance [36]. In lymphatic malignancies, the increased expression of miR-182 was found to be associated with drug resistance [37]. Wu et al. also revealed that miR-182 could negatively regulate PDCD4 expression to support cell-adhesion-mediated drug resistance of MM cells [23]. Consistently, our data also proved that miR-182 was highly expressed in MM patient serum, BMSCs, and BMSC-derived exosomes than those from BD patients. Moreover, we proved that exosomal miR-182 derived from MM-BMSCs could be internalized by MM cells, leading to an increase in miR-182 expression in MM cells, which resulted in enhanced MM cell metastasis and carfilzomib resistance. Furthermore, we first time revealed that BMSC-derived exosomal miR-182, serving as a crucial mediator in the BMSCs-MM cell crosstalk, induced the migration of MM cells and their resistance to carfilzomib. The results from this study provide further evidence to supported that exosomal miR-182 was a key target responsible for the progression of MM and the resistance to carfilzomib.

Hypoxia, acknowledged as a pivotal hallmark of cancer, is a primary catalyst for MM tumor growth and advancement [38]. HIF, composed of HIF-1α and HIF-2α, is considered the key controller of cellular responses to hypoxia and can contribute to an anti-apoptotic effect and facilitate drug resistance during tumor progression [39]. Previous studies have also shown that hypoxia can influence crosstalk between cells by enhancing exosome release and altering RNA/protein composition in exosomes [40]. For instance, hypoxia could enhance the release of exosomal miR-182 from BMSCs, thus promoting liver regeneration by inducing M2 macrophage polarization in vivo and in vitro [27]. Glioblastoma multiforme cells produced more exosomes and miR-185-5p was increased in the glioblastoma multiforme cell-derived exosomes under hypoxic conditions [19]. In agreement with the above reports, this study confirmed that miR-182 expression was upregulated in both BMSCs and BMSCs-Exos under hypoxic conditions. Further experiments described that HIF-1α directly interacted to miR-182 promoter, which was the important cause of miR-182 upregulation and exosomal secretion. Additionally, a few studies documented the regulatory mechanism between miR-182 and HIF-1α in different types of cells [41, 42]. The increased expression of miR-182-5p in nasopharyngeal carcinoma was partially induced by HIF-1α [42]. Another study by Li et al. demonstrated that miR-182 was activated during hypoxic conditions and formed a positive feedback loop with HIF-1α, thus promoting prostate cancer progression via targeting PHD2 and FIH1 [41]. Above all, our data firstly discovered the potential mechanism of the over-production of miR-182 in MM-BMSCs-Exos during hypoxia conditions.

SOX6, a member of the SOX family, is known to act as a tumor suppressor in many cancers and participates in the regulation of various malignant behaviors, such as cell proliferation and drug resistance and induction of apoptosis. For example, SOX6 expression was decreased in osteosarcoma and exerted a suppressive effect on migration, invasion, and epithelial-mesenchymal transition by regulating TWIST1 [43]. Mitogen-activated protein kinase kinase kinase kinase-4 (MAP4K4) was responsible for mediating the SOX6-induced autophagy, resulting in reduced chemosensitivity in cervical cancer [44]. Inhibition of SOX6 greatly lightened the capabilities of cell proliferation, metastasis and chemoresistance in cervical cancer [29]. In terms of the role of SOX6 in MM, it was not fully understood yet. Only one publication showed that miR-765 was upregulated in MM and promoted MM progression by directly targeting SOX6 [28]. In this study, it was discovered that miR-182 directly targeted the 3′-UTR of SOX6, resulting in its downregulation in MM cells. Inhibition of SOX6 effectively countered the inhibitory roles effects on tumor migration, invasion and drug resistance of MM cells mediated by the inhibition of miR-182 in BMSCs-Exos. These data indicate that dysregulation of SOX6 expression could play a crucial role in the pro-tumorigenic actions and drug resistance associated with miR-182 in MM.

## Conclusion

In conclusion, this study suggested that BMSC-derived exosomal miR-182 promoted the proliferation, metastasis, and carfilzomib resistance of MM cells by directly interacting with SOX6. These findings highlighted that BMSC-derived exosomal miR-182 might be a promising target for the improvement of carfilzomib resistance, and also provided valuable insights into the molecular mechanisms underlying MM. The major limitation is that the study primarily focused on in vitro experiments using cell lines. While cell line studies provide valuable insights, they may not fully replicate the complexity of the tumor microenvironment and interactions in vivo. Further investigations involving animal models or clinical samples will be essential to validate our findings.

## Data Availability

All data generated or analyzed are included in this article. Further inquiries can be directed to the corresponding author.

## Abbreviations

ANOVA: One-way analysis of variance
BD: Benign diseases
BM: Bone marrow
BMM: Bone marrow microenvironment
BMSCs: Bone marrow stromal cells
ChIP: Chromatin immunoprecipitation
EVs: Extracellular vesicles
FBS: Fetal bovine serum
MM: Multiple myeloma
miRNA: MicroRNA
MAP4K4: Mitogen-activated protein kinase kinase kinase kinase-4
NTA: Nanoparticle tracking analysis
PBS: Phosphate-buffered saline
PFA: Paraformaldehyde
RPMI: Rosewell Park Memorial Institute
RT-qPCR: Real-time polymerase chain reaction
SOX6: SRY-Box transcription factor 6
TEM: Transmission electron microscopy
